# 117 Evaluating Predicted vs. Actual Vancomycin Pharmacokinetic Target Attainment Using AUC Dosing

**DOI:** 10.1093/jbcr/irac012.119

**Published:** 2022-03-23

**Authors:** Allison N Boyd, Todd A Walroth, Christopher Bollinger, M B A Candidate, Brett C Hartman

**Affiliations:** Eskenazi Health, Indianapolis, Indiana; Eskenazi Health, Indianapolis, Indiana; Eskenazi Health, Indianapolis, Indiana; Richard M. Fairbanks Burn Center, Indianapolis, Indiana

## Abstract

**Introduction:**

Vancomycin is a broad-spectrum antibiotic commonly used in burn patients. Traditionally, vancomycin has been managed with a trough level dosing strategy. However, recent literature and practice changes have encouraged utilizing an area under the curve (AUC) dosing strategy. The objective of this study was to evaluate the likelihood of attaining therapeutic targets using population kinetics in burn patients utilizing AUC.

**Methods:**

This retrospective cohort included adult burn patients who received vancomycin dosed with an AUC strategy. Data collection covered 20 months following an institutional shift to AUC monitoring. Patients were excluded if they did not have pharmacokinetic (PK) data documented, received renal replacement therapy, or had a nonburn injury. The primary outcome was the difference between predicted and actual vancomycin AUC. Secondary outcomes included differences between additional PK parameters.

**Results:**

The AUC analysis included 14 patients. Mean (SD) age was 47 (12) years, 57% were male, and mean (SD) TBSA was 30 (21). The mean (SD) AUC in the predicted and actual groups was 522 (42) and 455 (122) mg*h/L, respectively (p=0.05). A total of 6 patients were evaluated for additional PK analysis, summarized in Table 1.

**Conclusions:**

Actual patient-specific AUC was significantly lower than predicted AUC. This corroborates previous trough-based data indicating burn patients dosed using standard population estimates are at risk of being subtherapeutic, likely due to their hypermetabolic state. Our findings suggest AUC monitoring is an appropriate dosing strategy for burn patients and potentially more accurate than a traditional trough-based approach.

